# The Effect of Holistic and Humanistic Approach on Professional Pride in Nursing Care

**DOI:** 10.1111/jan.70009

**Published:** 2025-06-16

**Authors:** Sebahat Kuşlu, Ayşe Eminoğlu, Mina Muşluoğlu

**Affiliations:** ^1^ Gaziantep Islam Science and Technology University of Health Sciences Department of Nursing Gaziantep Turkey; ^2^ Gaziantep City Hospital Gaziantep Turkey

**Keywords:** holistic nursing, humanistic approach, nursing competencies, professional pride

## Abstract

**Aim:**

This study examines the effect of nurses' holistic nursing competency levels and humanistic behaviour skills on their professional pride levels.

**Design:**

The study was designed as a cross‐sectional study.

**Methods:**

The sample consisted of 224 nurses working in a city hospital. The data were collected using the Personal Information Form, the Holistic Nursing Competence Scale, the Humanistic Behaviour Skill Scale in Nursing Practices and the Pride in Nursing Profession Scale. Independent groups *t*‐test, ANOVA, and regression analyses were applied to analyse the data.

**Results:**

The mean ± standard deviation of the total score of the nurses participating in the study from the Holistic Nursing Competence Scale was 170.48 ± 42.41, the mean ± standard deviation of the total score from the Humanistic Behaviour in Nursing Practice Scale was 121.35 ± 20.81, and the mean ± standard deviation of the total score from the Pride in Nursing Profession Scale was 79.65 ± 22.80. A statistically significant positive relationship was found between the Holistic Nursing Competence Scale and the Humanistic Behaviour in Nursing Practice Scale. The results showed a statistically significant relationship between the Holistic Nursing Competence Scale, the Humanistic Behaviour in Nursing Practice Scale and the Pride in Nursing Profession Scale. In addition, it was determined that nurses' holistic and humanistic behaviour skills explained 12.2% of the change in professional pride. The holistic nursing competencies and professional pride levels of nurses who willingly chose the nursing profession and loved their profession were found to be statistically significantly higher (*p* < 0.05).

**Conclusion:**

Nurses demonstrated above‐average holistic and humanistic competencies, yet their professional pride remained below average. This indicates that professional pride may be shaped not only by individual skills but also by external factors such as working conditions and societal perceptions of the profession.

**Implication for the Profession and/or Patient Care:**

These findings suggest that to increase professional pride among nurses, not only is the development of individual competencies insufficient, but also the improvement of workplace environments and greater societal appreciation of the profession are necessary. Without addressing systemic and societal challenges, the development of holistic and humanistic nursing competences may have a limited impact on professional satisfaction. On the other hand, nurses' provision of care services in line with holistic and humanistic principles positively affects their professional satisfaction and the quality of patient care, patient satisfaction, safety and general health outcomes.

**Reporting Method:**

This study adhered to the STROBE criteria.

**Summary**

- What is already known about this topic?
  ○ The ability to act humanistically in nursing care affects the way care is delivered.
  ○ Holistic nursing skills are important in the delivery of humanistic care.
  ○ Nurses' perceptions of professional pride also directly affect the delivery of care.
- What this paper adds?
  ○ There is a lack of studies investigating the socio‐demographic characteristics of nurses and their holistic and humanistic behaviour skills.
  ○ There is a lack of studies that reveal the relationship between holistic and humanistic behavioural skills of nurses.
  ○ There is no study to reveal the effect of nurses' holistic and humanistic behavioural skills on the level of professional pride.

## 1 Introduction

Human is a whole with biological, psychological, social and cultural aspects (Bayindir and Bicer 2019). These dimensions constantly interact, forming an inseparable whole (Ay 2021). In the care processes, the most critical and complex part of nursing services, the individual's physical, mental and spiritual aspects, should be considered as a whole, and care should be provided in this direction (Riegel et al. 2021). The nursing profession provides an individual‐oriented service with a theory‐based, communication and interaction‐based, holistic approach. The holistic nursing approach considers the individual's physical health and psychosocial needs and supports the nurse's personal and professional development (PD) in this process (Ozdemir and Agacdiken 2022).

### 1.1 Background

The humanistic approach, which forms the basis of the nursing profession, includes a productive, therapeutic and effective communication process based on the autonomy of the individual (Yalcin and Asti 2011). Nursing is considered a ‘science’ with the advancement of science and technology, and it is also recognised as one of the most reliable professions (Stecker 2016). However, for nurses to strengthen their professional identity and social credibility, they must develop professional autonomy, ethical responsibility and altruism (Cruess et al. 2016).

Professional pride is closely related to nurses' job satisfaction, professionalism, professional competence and responsibilities (Jeon et al. 2020). However, today's intense workload, stressful working conditions and the perception of the nursing profession in society may cause professional pride not to be high enough. This may both negatively affect the professional satisfaction of nurses and lead to a decrease in the quality of care. Professional pride is an essential factor that increases individual satisfaction and the value of nursing in the social sense (Badran and Mohamed 2024). Although the relationship between professional pride and concepts such as job satisfaction and professionalism has been examined in the literature, there is a limited number of studies on its relationship with humanistic approaches and holistic nursing skills.

### 1.2 Aims

This study aims to fill the literature gap and reveal how nurses' professional pride levels affect their holistic nursing and humanistic behaviour skills.

In this study, answers to the following questions were sought;

1. Is there a difference between holistic nursing competencies, humanistic behaviour skills and professional pride levels of nurses in terms of their socio‐demographic characteristics?
2. How are the holistic nursing competencies, humanistic behaviour skills and professional pride levels of nurses?
3. What is the relationship between nurses' holistic nursing competencies, humanistic behaviour skills and professional pride levels?
4. How do nurses' holistic nursing competencies and humanistic behaviour skills affect their professional pride levels?

## 2 Materials and Methods

### 2.1 Type of Research

This research was designed as a descriptive type, one of the cross‐sectional research methods.

### 2.2 Population and Sample of the Study

The study population consisted of nurses working in Gaziantep City Hospital in Turkey. The G*Power 3.1.9.4 program was used to calculate the sample size. Previous studies were examined (Yilmaz et al. 2022; Aydin et al. 2023), and the calculation was made with a *t*‐test. By determining the expected confidence intervals of the ‘Holistic Nursing Competence Scale (HNCS)’, the confidence interval *α* = 0.01, the power of the test (1 − *β*) 0.99 and the effect size *d* = 0.3457944 were calculated for a total of 184 people. The sample consisted of 224 nurses who volunteered to participate in this study.

Inclusion criteria:

- Working as a nurse in the city hospital where the study was conducted.
- Being over 18 years of age.
- Voluntarily accept participation in the study.

### 2.3 Data Collection

The research was conducted between 01.04.2024 and 01.08.2024, and the data were collected through a face‐to‐face questionnaire. This period was determined as a time period in which the participants could spare more time for the questionnaire application, taking into account the working order and staff density of the hospital. This form consists of a Personal Information Form (14 questions), a HNCS, a Humanistic Behaviour Skill Scale in Nursing Practices (HBSSNP) and a Pride in Nursing Profession Scale (PNPS). After the nurses were informed about the study's purpose, scope and inclusion criteria, the questionnaire form was completed after obtaining their verbal and written consent.

### 2.4 Data Collection Tools

In this study, both continuous (numerical) and categorical variables were collected. Continuous variables included age (years), total work experience (years), weekly working hours (hours) and scale scores (points). Categorical variables included gender (male, female), income status (less than expenditure, equals expenditure, more than expenditure), education level (Health vocational high school, island licences, undergraduate, postgraduate), the status of voluntary choice of nursing profession (yes, no), mode of operation (day, night, night and day), occupation affection status (yes, no) and worked services (intensive care, international service, surgical services, emergency service, polyclinic).

#### 2.4.1 Personal Information Form

This form consists of 14 questions and includes open‐ended and multiple‐choice questions. The form includes questions about nurses' age, gender, marital status, income status, number of children, educational status, years of working as a nurse, willingly choosing the profession, working style, liking the profession, weekly working time, the service they work in, handling patients as a whole and effective and healthy communication with patients.

#### 2.4.2 Holistic Nursing Competence Scale

HNCS developed by Takase and Teraoka (2011). The Turkish adaptation study was conducted by Aydin and Hicdurmaz (2019). The scale comprises 36 items and two sections and is a 7‐point Likert‐type scale. The first part (A) includes the ‘General Ability (GA)’ subscale and consists of questions about usual behaviours as a person, not as a nurse. The items in this section, which consist of 7 questions, are graded from never: 1 to constantly: 7. The second part (B) measures competence as a nurse. It consists of five subscales named ‘Personnel Education and Management (PEM)’, ‘Ethically Oriented Practice (EOP)’, ‘Nursing Care in the Team (NCT)’, and ‘PD’. Consisting of 29 items, this section (B) is graded from insufficient: 1 to very sufficient: 7. There are no reverse‐scored items or cut‐off points in the scale. Cronbach's alpha value of the scale is between 0.67 and 0.91 for sub‐dimensions and the total. The range of points that can be obtained from the scale is 36–252. As the score obtained from the scale increases, holistic nursing competence also increases (Aydin and Hicdurmaz 2019). In this study, the Cronbach's alpha coefficient was calculated as 0.984.

#### 2.4.3 Humanistic Behaviour Skill Scale in Nursing Practice

The scale, whose Turkish adaptation study was conducted by Yanmis et al. (2022), consists of 5 sub‐dimensions (nursing communication skill [NCS], psychological adaptation skill [PAS], ethical and legal issues application skill [ELAS], aesthetic nursing skill [ANS], practical care skill [PCS]). The scale has 29 questions, and the answer option is a 5‐point Likert type. The scoring of the scale is between Very Appropriate: 5 and Not Appropriate at All: 1. The scale's total score is calculated by summing the scores obtained from the items. There are no reverse‐coded items in the scale. While the Cronbach alpha value for the overall scale is 0.93, it varies between 0.71 and 0.89 for the subscales. The lowest score that can be obtained from the scale is 29, and the highest score is 145. Sub‐dimension scores are calculated in the same way. High scores indicate the ability to act humanistically in nursing practices is also high (Yanmis et al. 2022). In this study, the Cronbach's alpha coefficient of the scale was calculated as 0.974.

#### 2.4.4 Profession Nursing Pride Scale

The scale, which was adapted into Turkish by Aydin et al. (2022), consists of 27 items and 5 sub‐dimensions (professional emotion [PE], role satisfaction [RS], problem‐solving role [PSR], self‐achievement [SA], desire to continue the profession [DCP]). The scoring of the scale is in the range of Strongly Agree: 5 to Strongly Disagree: 1 and is in a 5‐point Likert‐type scale. Cronbach's alpha value for the overall scale is 0.89. The score that can be obtained from the scale is between 27 and 135. As the score increases, the pride of nursing professionals in their profession increases (Aydin et al. 2022). In this study, the Cronbach's alpha coefficient for the total scale was calculated as 0.957.

### 2.5 Analysis and Evaluation of Data

The SPSS (Statistical Packages for the Social Sciences) Version 22 program analysed the data. Personal information about the nurses was given in numbers and percentages. Number, percentage, arithmetic mean, standard deviation, minimum and maximum values were used to evaluate the scores obtained from the scales. ‘XX ± YY’, ‘XX’ represents the arithmetic mean of the measured variable and ‘YY’ represents its standard deviation. Kolmogorov–Smirnov tests, Skewness and Kurtosis values, histogram graph, coefficient of variation and detrended graph evaluate the normal distribution of the data. In addition, it is stated in the literature that Skewness and Kurtosis values between ± 1.0 are sufficient for normal distribution (Hair et al. 2010). In this study, Skewness: −0.319 Kurtosis: 0.110 for the HNCS; Skewness: −0.236 Kurtosis: −0.561 for the Humanistic Behaviour in Nursing Practices Scale; Skewness: −0.236 Kurtosis: −0.561 for the PNPS: −0.236 Kurtosis: −0.561; for the PNPS Skewness: −0.347 Kurtosis: −0.263 and it was found that the variables appeared to be approximately normally distributed. Since skewness and kurtosis values, histogram graphs and detrended graphs were provided from normality criteria, it was determined that the data were normally distributed. In this direction, a student *t*‐test was used for categorical variables with two groups, an ANOVA Test for categorical variables with three or more groups, Pearson Correlation Analysis was used to determine the relationship between the scales, and multiple regression analysis was used to evaluate the effect of variables. The significance level was taken as *p* < 0.05.

### 2.6 Ethical Aspects of the Research

Approval was obtained from the Gaziantep Islam Science and Technology University Non‐Interventional Clinical Research Ethics Committee for the conduct of the study (Decision Date: 20.07.2023, Decision No: 276.27.07). During the entire research process, the principles of research and publication ethics by the Principles of the Declaration of Helsinki were complied with. In addition, data were collected after the nurses were informed about the purpose and scope of the study and their informed voluntary consent was obtained.

## 3 Findings

### 3.1 Characteristics of the Sample

The mean age ± standard deviation of the nurses was 26.35 ± 4.00 years, 55.8% were female, 76.3% were single, 43.8% had income equal to expenses, 89.3% had bachelor's degrees, and the mean number of children ± standard deviation was 0.19 ± 0.63 (Table 1). It was found that 64.7% of the nurses chose the nursing profession willingly, 43.3% worked during the day, 67.4% enjoyed their profession, 42.9% worked in internal services, the mean years of employment in nursing ± standard deviation was 3.19 ± 3.23 and the mean weekly working hours ± standard deviation was 47.09 ± 10.21 h (Table 2). 93.3% of the nurses stated that they treated the individuals they cared for as a whole in terms of bio‐psycho‐socio‐cultural aspects without discriminating against religion, language and race, and 96% said that they established effective and healthy communication with the individuals they cared for (Table 3).

**TABLE 1:** The correlation between socio‐demographic characteristics of nurses and total and subscale scores of HNCS, HBSSNP, PNPS.

Variables		Gender		Test and significance value	Marital status		Test and significance value	Income status			Test and significance value	Education status				Test and significance value
		Woman	Male		Married	Single		Income less than expenditure	Income equals expenditure	Income more than expenditure		Health vocational high school	Island licences	Undergraduate	Postgraduate	
		125 (55.8)	99 (44.2)		53 (23.7)	171 (76.3)		106 (47.3)	98 (43.8)	20 (8.9)		13 (5.8)	5 (2.2)	200 (89.3)	6 (2.7)	
HNCS	GA	34.71 ± 7.70	34.83 ± 9.53	*t*: −0.107 *p*: 0.915	33.88 ± 8.36	35.04 ± 8.60	*t*: −0.859 *p*: 0.391	34.41 ± 8.77	34.40 ± 8.33	38.40 ± 7.81	*F*: 2.004 *p*: 0.137	34.46 ± 10.88	36.60 ± 12.36	34.59 ± 8.30	39.83 ± 7.96	*F*: 0.813 *p*: 0.488
	PEM	38.71 ± 11.33	41.33 ± 11.34	*t*: −1.718 *p*: 0.087	40.28 ± 11.52	39.74 ± 11.38	*t*: 0.301 *p*: 0.764	39.14 ± 11.71	40.50 ± 10.53	40.65 ± 13.86	*F*: 0.411 *p*: 0.663	36.46 ± 14.72	46.00 ± 11.57	39.74 ± 11.14	46.33 ± 10.01	*F*: 1.533 *p*: 0.207
	EOP	42.88 ± 11.66	44.14 ± 12.39	*t*: −0.782 *p*: 0.435	42.16 ± 12.39	43.83 ± 11.86	*t*: −0.881 *p*: 0.379	42.71 ± 13.06	44.38 ± 10.67	42.60 ± 12.36	*F*: 0.546 *p*: 0.580	41.61 ± 17.26	46.40 ± 15.75	43.31 ± 11.60	49.16 ± 8.63	*F*: 0.664 *p*: 0.575
	NCT	32.80 ± 9.59	34.23 ± 9.51	*t*: −1.107 *p*: 0.270	32.54 ± 9.83	33.71 ± 9.49	*t*: −0.775 *p*: 0.439	33.16 ± 10.38	33.63 ± 8.55	33.95 ± 10.22	*F*: 0.093 *p*: 0.911	31.15 ± 13.69	36.00 ± 12.70	33.37 ± 9.24	38.50 ± 7.23	*F*: 0.929 *p*: 0.428
	PD	18.65 ± 5.43	19.37 ± 5.79	*t*: −0.953 *p*: 0.342	18.47 ± 6.01	19.12 ± 5.47	*t*: −0.746 *p*: 0.457	18.61 ± 6.07	19.21 ± 5.10	19.70 ± 5.45	*F*: 0.476 *p*: 0.622	17.61 ± 7.08	21.20 ± 7.69	18.92 ± 5.46	21.83 ± 4.83	*F*: 1.047 *p*: 0.373
	HNCS total	167.76 ± 40.97	173.91 ± 44.14	*t*: −1.078 *p*: 0.282	167.35 ± 43.84	171.45 ± 42.05	*t*: −0.614 *p*: 0.540	168.04 ± 45.95	172.14 ± 38.01	175.30 ± 44.52	*F*: 0.377 *p*: 0.687	161.30 ± 61.89	186.20 ± 58.93	169.93 ± 40.73	195.66 ± 31.74	*F*: 1.150 *p*: 0.330
HBSSNP	NCS	28.16 ± 4.30	27.70 ± 5.63	*t*: 0.662 *p*: 0.509	27.86 ± 4.35	27.98 ± 5.10	*t*: −0.155 *p*: 0.877	27.63 ± 5.38	27.98 ± 4.26	29.55 ± 5.37	*F*: 1.280 *p*: 0.280	28.53 ± 3.82	30.40 ± 1.81	27.79 ± 5.06	30.33 ± 3.07	*F*: 1.011 *p*: 0.389
	PAS	15.66 ± 2.94	15.81 ± 3.54	*t*: −0.356 *p*: 0.723	15.67 ± 3.13	15.74 ± 3.25	*t*: −0.137 *p*: 0.891	15.62 ± 3.39	15.60 ± 3.07	16.95 ± 2.81	*F*: 1.583 *p*: 0.208	16.38 ± 2.32	16.80 ± 2.16	15.60 ± 3.29	17.66 ± 2.42	*F*: 1.192 *p*: 0.314
	ELAS	29.28 ± 4.57	27.95 ± 6.20	*t*: 1.780 *p*: 0.077	28.18 ± 5.10	28.85 ± 5.47	*t*: −0.792 *p*: 0.429	28.24 ± 5.86	29.13 ± 4.88	29.00 ± 5.14	*F*: 0.723 *p*: 0.487	30.69 ± 4.69	29.60 ± 3.78	28.45 ± 5.48	32.00 ± 2.60	*F*: 1.544 *p*: 0.204
	ANS	16.12 ± 2.86	14.90 ± 3.81	***t*: 2.627** ***p*: 0.009**	14.94 ± 3.04	15.78 ± 3.44	*t*: −1.595 *p*: 0.112	15.32 ± 3.42	15.73 ± 3.32	16.25 ± 3.22	*F*: 0.814 *p*: 0.444	16.69 ± 2.17	17.60 ± 2.07	15.42 ± 3.45	16.83 ± 2.22	*F*: 1.505 *p*: 0.214
	PCS	32.84 ± 9.50	34.05 ± 9.57	*t*: −0.937 *p*: 0.350	32.41 ± 10.03	33.67 ± 9.38	*t*: −0.842 *p*: 0.401	32.89 ± 10.16	33.67 ± 8.84	34.50 ± 9.70	*F*: 0.319 *p*: 0.727	30.76 ± 12.70	36.60 ± 13.35	33.31 ± 9.22	38.66 ± 8.54	*F*: 1.136 *p*: 0.336
	HBSSNP Total	122.08 ± 18.85	120.44 ± 23.11	*t*: 0.570 *p*: 0.570	119.09 ± 21.54	122.05 ± 20.59	*t*: −0.906 *p*: 0.366	119.71 ± 22.27	122.13 ± 19.23	126.25 ± 20.25	*F*: 0.949 *p*: 0.389	123.07 ± 19.53	131.00 ± 18.19	120.58 ± 21.05	135.50 ± 11.32	*F*: 1.412 *p*: 0.240
PNPS	PE	18.05 ± 4.94	17.24 ± 5.59	*t*: 1.154 *p*: 0.250	17.37 ± 4.96	17.79 ± 5.33	*t*: −0.506 *p*: 0.613	16.70 ± 5.57	18.48 ± 4.61	19.05 ± 5.66	***F*: 3.762** ***p*: 0.025** **(2 > 1)**	16.23 ± 6.40	19.20 ± 5.71	17.67 ± 5.19	20.50 ± 3.27	*F*: 1.049 *p*: 0.372
	RS	15.97 ± 5.19	15.51 ± 6.15	*t*: 0.595 *p*: 0.552	14.77 ± 5.22	16.08 ± 5.73	*t*: −1.481 *p*: 0.140	15.07 ± 5.94	16.19 ± 4.94	17.40 ± 6.78	*F*: 1.935 *p*: 0.147	15.46 ± 5.63	18.00 ± 4.47	15.70 ± 5.66	17.00 ± 6.44	*F*: 0.376 *p*: 0.770
	PSR	20.24 ± 5.54	18.76 ± 6.60	*t*: 1.776 *p*: 0.077	19.43 ± 5.94	19.63 ± 6.12	*t*: −0.213 *p*: 0.832	18.56 ± 6.70	20.56 ± 5.24	20.25 ± 5.66	***F*: 2.931** ***p*: 0.055 (2 > 1)**	18.61 ± 7.70	18.60 ± 6.54	19.55 ± 6.01	23.66 ± 1.63	*F*: 1.061 *p*: 0.366
	SA	11.88 ± 4.09	11.27 ± 4.75	*t*: 1.016 *p*: 0.311	10.67 ± 3.81	11.90 ± 4.47	*t*: −1.793 *p*: 0.074	10.90 ± 4.74	12.13 ± 3.79	12.80 ± 4.33	*F*: 2.887 *p*: 0.058	10.84 ± 5.04	12.80 ± 5.40	11.58 ± 4.31	13.16 ± 3.97	*F*: 0.512 *p*: 0.674
	DCP	15.68 ± 4.84	14.11 ± 5.77	***t*: 2.167** ***p*: 0.031**	13.98 ± 4.95	15.29 ± 5.40	*t*: −1.580 *p*: 0.115	14.16 ± 5.80	15.40 ± 4.62	17.30 ± 5.13	***F*: 3.558** ***p*: 0.030** **(3 > 1)**	13.76 ± 6.20	15.00 ± 6.08	15.03 ± 5.26	16.00 ± 5.47	*F*: 0.302 *p*: 0.824
	PNPS total	81.83 ± 21.02	76.90 ± 24.70	*t*: 1.610 *p*: 0.109	76.24 ± 20.40	80.71 ± 23.45	*t*: −1.248 *p*: 0.213	75.41 ± 25.13	82.78 ± 19.04	86.80 ± 23.54	***F*: 3.833** ***p*: 0.023**	74.92 ± 29.40	83.60 ± 24.92	79.54 ± 22.49	90.33 ± 16.29	*F*: 0.674 *p*: 0.569
Age: 26.35 ± 4.00								Number of children: 0.19 ± 0.63								

*Note:* *F*: one way Anova test value, *t*: independent sample *T* test value, *p*: significance level, XX ± YY: mean ± standard deviation. Significant *p* values are shown in bold, *p <* 0.05.

Abbreviations: ANS, aesthetic nursing skill; DCP, Desire to Continue the Profession; ELAS, ethical and legal issues application skill; EOP, Ethically Oriented Practice; GA, General Ability; HBSSNP, Humanistic Behaviour Skill Scale in Nursing Practice; HNCS, Holistic Nursing Competence Scale; NCS, nursing communication skill; NCT, Nursing Care in the Team; PAS, psychological adaptation skill; PCS, practical care skill; PD, professional development; PE, professional emotion; PEM, Personnel Education and Management; PNPS, Profession Nursing Pride Scale; PSR, problem‐solving role; RS, role satisfaction; SA, self‐achievement.

**TABLE 2:** The correlation between nurses' occupational characteristics and total and subscale scores of HNCS, HBSSNP, PNPS.

Variables		Status of voluntary choice of nursing profession		Test and significance value	Mode of operation			Test and significance value	Occupation affection status		Test and significance value	Worked service					Test and significance value
		Yes	No		Daytime	Night	Night and day		Yes	No		Intensive care	Internal service	Surgical service	Emergency service	Polyclinic	
		145 (64.7)	79 (35.3)		97 (43.3)	84 (37.5)	43 (19.2)		151 (67.4)	73 (32.6)		60 (26.8)	96 (42.9)	42 (18.8)	3 (1.3)	23 (10.3)	
HNCS	GA	35.79 ± 8.57	32.88 ± 8.20	***t*: 2.462** ***p*: 0.015**	32.93 ± 8.58	35.98 ± 8.31	36.51 ± 8.27	***F*: 4.090** ***p*: 0.018 (2 > 1)**	36.22 ± 8.32	31.75 ± 8.24	***t*: 3.781** ***p* < 0.001**	36.46 ± 7.93	34.21 ± 9.56	34.26 ± 7.44	37.00 ± 5.19	33.26 ± 7.52	*F*: 0.959 *p*: 0.431
	PEM	41.17 ± 11.04	37.46 ± 11.68	***t*: 2.353** ***p*: 0.019**	38.26 ± 11.14	40.65 ± 11.11	41.95 ± 12.23	*F*: 1.893 *p*: 0.153	41.45 ± 11.28	36.58 ± 10.96	***t*: 3.053** ***p*: 0.003**	42.23 ± 10.23	38.89 ± 12.59	39.19 ± 10.37	41.66 ± 11.59	38.78 ± 10.69	*F*: 0.928 *p*: 0.448
	EOP	44.85 ± 11.52	40.83 ± 12.43	***t*: 2.424** ***p*: 0.016**	40.54 ± 11.85	45.82 ± 11.37	45.30 ± 12.31	***F*: 5.192** ***p*: 0.006** **(2 > 1)**	44.92 ± 11.53	40.36 ± 12.39	***t*: 2.701** ***p*: 0.007**	45.50 ± 10.64	43.12 ± 13.63	43.80 ± 10.07	45.00 ± 10.14	38.47 ± 10.61	*F*: 1.481 *p*: 0.209
	NCT	34.62 ± 9.25	31.25 ± 9.80	***t*: 2.553** ***p*: 0.011**	31.42 ± 9.56	34.83 ± 9.18	35.25 ± 9.67	***F*: 3.921** ***p*: 0.021** **(2 > 1)**	34.69 ± 9.14	30.83 ± 9.94	***t*: 2.875** ***p*: 0.004**	35.33 ± 8.30	33.50 ± 10.81	33.00 ± 7.85	32.33 ± 4.50	29.17 ± 9.56	*F*: 1.788 *p*: 0.132
	PD	19.60 ± 5.34	17.82 ± 5.89	***t*: 2.292** ***p*: 0.023**	17.79 ± 5.59	19.78 ± 5.43	20.04 ± 5.54	***F*: 3.928** ***p*: 0.021** **(2 > 1)**	19.73 ± 5.39	17.39 ± 5.72	***t*: 2.981** ***p*: 0.003**	19.96 ± 4.99	18.67 ± 6.46	19.09 ± 4.38	19.00 ± 3.60	17.39 ± 5.23	*F*: 1.004 *p*: 0.407
	HNCS Total	176.05 ± 40.67	160.26 ± 43.88	***t*: 2.699** ***p*: 0.007**	160.96 ± 42.95	177.08 ± 40.73	179.06 ± 41.04	***F*: 4.473** ***p*: 0.012 (1 < 2) (1 < 3)**	177.03 ± 40.51	156.94 ± 43.33	***t*: 3.400** ***p* < 0.001**	179.50 ± 36.42	168.41 ± 49.04	169.35 ± 34.94	175.00 ± 33.95	157.08 ± 38.49	*F*: 1.332 *p*: 0.259
HBSSNP	NCS	28.42 ± 4.44	27.10 ± 5.63	*t*: 1.807 *p*: 0.073	27.86 ± 4.92	27.73 ± 4.76	28.60 ± 5.31	*F*: 0.468 *p*: 0.627	28.57 ± 4.64	26.68 ± 5.27	***t*: 2.730** ***p*: 0.007**	27.35 ± 5.00	28.75 ± 5.21	28.02 ± 4.12	27.00 ± 3.46	26.26 ± 4.67	*F*: 1.576 *p*: 0.182
	PAS	16.03 ± 2.94	15.17 ± 3.61	*t*: 1.807 *p*: 0.073	15.49 ± 3.26	15.76 ± 3.06	16.20 ± 3.41	*F*: 0.739 *p*: 0.479	16.17 ± 2.99	14.80 ± 3.48	***t*: 2.887** ***p*: 0.005**	15.73 ± 3.17	16.18 ± 3.28	15.61 ± 3.09	15.33 ± 1.52	14.08 ± 3.10	*F*: 2.047 *p*: 0.089
	ELAS	29.30 ± 4.80	27.59 ± 6.19	***t*: 2.127** ***p*: 0.035**	28.21 ± 5.33	29.03 ± 5.28	29.13 ± 5.75	*F*: 0.695 *p*: 0.500	29.50 ± 4.95	27.02 ± 5.88	***t*: 3.303** ***p*: 0.001**	28.21 ± 5.68	29.33 ± 5.35	29.59 ± 4.42	30.66 ± 1.15	25.43 ± 5.63	***F*: 3.064** ***p*: 0.018 (2 > 5) (3 > 5)**
	ANS	15.79 ± 3.15	15.20 ± 3.71	*t*: 1.198 *p*: 0.233	15.28 ± 3.50	15.64 ± 3.15	16.13 ± 3.43	*F*: 0.973 *p*: 0.379	16.00 ± 3.20	14.71 ± 3.54	***t*: 2.644** ***p*: 0.009**	15.56 ± 3.12	16.05 ± 3.41	15.69 ± 2.91	15.00 ± 1.73	13.56 ± 4.07	***F*: 2.646** ***p*: 0.034 (2 > 5)**
	PCS	34.48 ± 9.26	31.35 ± 9.74	***t*: 2.370** ***p*: 0.019**	31.39 ± 9.38	34.69 ± 9.33	35.30 ± 9.63	***F*: 3.874** ***p*: 0.022**	34.66 ± 9.30	30.71 ± 9.50	***t*: 2.961** ***p*: 0.003**	35.15 ± 8.50	33.03 ± 10.92	33.50 ± 7.65	32.33 ± 4.93	30.13 ± 8.96	*F*: 1.233 *p*: 0.298
	HBSSNP Total	124.04 ± 18.86	116.43 ± 23.30	***t*: 2.492** ***p*: 0.014**	118.25 ± 21.27	122.86 ± 19.65	125.39 ± 21.40	*F*: 2.128 *p*: 0.122	124.94 ± 19.88	113.94 ± 20.86	***t*: 3.817** ***p* < 0.001**	122.01 ± 20.91	123.35 ± 22.17	122.42 ± 16.91	120.33 ± 11.93	109.47 ± 19.46	*F*: 2.184 *p*: 0.072
PNPS	PE	18.59 ± 4.99	16.05 ± 5.32	***t*: 3.556** ***p* < 0.001**	17.52 ± 5.42	17.50 ± 4.91	18.46 ± 5.50	*F*: 0.570 *p*: 0.566	18.56 ± 5.02	15.89 ± 5.26	***t*: 3.683** ***p* < 0.001**	17.71 ± 4.64	17.25 ± 5.98	18.78 ± 3.76	18.66 ± 9.29	17.39 ± 5.36	*F*: 0.668 *p*: 0.615
	RS	16.23 ± 5.74	14.92 ± 5.36	*t*: 1.670 *p*: 0.096	14.43 ± 4.85	16.05 ± 5.52	18.23 ± 6.61	***F*: 7.342** ***p* < 0.001** **(3 > 1)**	16.32 ± 5.57	18.17 ± 6.81	***t*: 2.126** ***p*: 0.035**	16.15 ± 5.23	15.39 ± 6.58	16.69 ± 3.63	18.33 ± 9.60	14.34 ± 4.72	*F*: 0.975 *p*: 0.422
	PSR	20.37 ± 5.70	18.13 ± 6.47	***t*: 2.675** ***p*: 0.008**	18.57 ± 6.36	20.28 ± 5.50	20.51 ± 6.24	*F*: 2.428 *p*: 0.091	20.27 ± 5.57	18.17 ± 6.81	***t*: 2.282** ***p*: 0.024**	20.51 ± 4.94	19.04 ± 7.28	21.21 ± 3.99	18.33 ± 4.72	16.65 ± 5.39	***F*: 2.761** ***p*: 0.029 (3 > 5)**
	SA	12.23 ± 4.33	10.46 ± 4.18	***t*: 2.950** ***p*: 0.004**	10.91 ± 4.43	11.95 ± 4.08	12.51 ± 4.53	*F*: 2.439 *p*: 0.090	12.36 ± 4.26	10.05 ± 4.13	***t*: 3.833** ***p* < 0.001**	11.30 ± 4.23	11.65 ± 4.76	12.19 ± 3.50	17.66 ± 2.51	10.39 ± 3.90	*F*: 2.213 *p*: 0.069
	DCP	15.51 ± 5.40	14.02 ± 5.05	***t***: **2.010** ***p***: **0.046**	14.35 ± 4.93	15.05 ± 5.22	16.27 ± 6.16	*F*: 1.988 *p*: 0.139	15.66 ± 5.19	13.57 ± 5.33	***t***: **2.803** ***p***: **0.006**	14.23 ± 4.73	15.07 ± 6.11	16.28 ± 4.06	17.00 ± 8.88	13.95 ± 4.53	*F*: 1.263 *p*: 0.286
	PNPS Total	82.95 ± 22.12	73.60 ± 22.93	***t*: 2.982** ***p*: 0.003**	75.80 ± 21.98	80.85 ± 21.28	86.00 ± 26.12	***F*: 3.228** ***p*: 0.042** **(3 > 1)**	83.19 ± 21.93	72.32 ± 22.97	***t*: 3.423** ***p*: < 0.001**	79.91 ± 19.55	78.41 ± 27.37	85.16 ± 15.97	90.00 ± 23.64	72.73 ± 18.82	*F*: 1.379 *p*: 0.242
Years of working as a nurse: 3.19 ± 3.23									Weekly working time: 47.09 ± 10.21								

*Note:* *F*: one way Anova test value, *t*: independent sample *T* test value, *p*: significance level, XX ± YY: mean ± standard deviation. Significant *p* values are shown in bold, *p* < 0.05.

Abbreviations: ANS, aesthetic nursing skill; DCP, Desire to Continue the Profession; ELAS, ethical and legal issues application skill; EOP, Ethically Oriented Practice; GA, General Ability; HBSSNP, Humanistic Behaviour Skill Scale in Nursing Practice; HNCS, Holistic Nursing Competence Scale; NCS, nursing communication skill; NCT, Nursing Care in the Team; PAS, psychological adaptation skill; PCS, practical care skill; PD, professional development; PE, professional emotion; PEM, Personnel Education and Management; PNPS, Profession Nursing Pride Scale; PSR, problem‐solving role; RS, role satisfaction; SA, self‐achievement.

**TABLE 3:** The correlation between nurses' views on holistic and humanistic approach and total and subscale scores of HNCS, HBSSNP, PNPS.

Variables		Considering the cared individual as a whole in terms of bio‐psycho‐psycho‐socio‐cultural aspects without discriminating religion, language, race		Test and significance value	Effective and healthy communication with the individual being cared for		Test and significance value
		Yes	No		Yes	No	
		209 (93.3)	15 (6.7)		215 (96.0)	9 (4.0)	
HNCS	GA	35.14 ± 8.25	29.46 ± 10.79	***t***: **2.518**; ***p***: **0.012**	35.17 ± 8.21	25.00 ± 10.79	***t***: **3.595**; ***p* < 0.001**
	PEM	40.22 ± 11.11	35.00 ± 14.26	*t*: 1.722; *p*: 0.086	40.21 ± 11.14	31.66 ± 14.72	***t***: **2.225**; ***p***: **0.027**
	EOP	43.85 ± 11.83	37.60 ± 12.97	***t***: **1.965**; ***p***: **0.051**	43.96 ± 11.78	30.77 ± 9.89	***t***: **3.306**; ***p***: **0.001**
	NCT	33.81 ± 9.34	28.13 ± 11.35	***t***: **2.243**; ***p***: **0.026**	33.94 ± 9.19	21.22 ± 10.75	***t***: **4.041**; ***p* < 0.001**
	PD	19.21 ± 5.51	15.66 ± 5.87	***t***: **2.393**; ***p***: **0.018**	19.23 ± 5.50	12.66 ± 4.18	***t***: **3.537**; ***p* < 0.001**
	HNCS total	172.25 ± 41.23	145.86 ± 51.94	***t***: **2.351**; ***p***: **0.020**	172.54 ± 41.04	121.33 ± 47.61	***t***: **3.645**; ***p* < 0.001**
HBSSNP	NCS	28.03 ± 4.88	26.86 ± 5.52	*t*: 0.889; *p*: 0.375	28.00 ± 4.96	27.00 ± 4.15	*t*: 0.595; *p*: 0.552
	PAS	15.89 ± 3.19	13.53 ± 2.69	***t***: **2.782**; ***p***: **0.006**	15.81 ± 3.23	13.66 ± 1.93	***t***: **1.979**; ***p***: **0.049**
	ELAS	28.86 ± 5.39	26.40 ± 4.91	*t*: 1.720; *p*: 0.087	28.81 ± 5.37	26.00 ± 5.22	*t*: 1.540; *p*: 0.125
	ANS	15.70 ± 3.34	13.93 ± 3.26	***t***: **1.982**; ***p***: **0.049**	15.65 ± 3.33	13.88 ± 3.78	*t*: 1.549; *p*: 0.123
	PCS	33.80 ± 9.30	27.40 ± 10.99	***t***: **2.545**; ***p***: **0.012**	33.87 ± 9.25	21.55 ± 8.97	***t***: **3.918**; ***p* < 0.001**
	HBSSNP total	122.30 ± 20.46	108.13 ± 21.85	***t***: **2.580**; ***p***: **0.011**	122.16 ± 20.64	102.11 ± 15.32	***t***: **2.877**; ***p***: **0.004**
PNPS	PE	17.70 ± 5.32	17.53 ± 4.10	*t*: 0.124; *p*: 0.901	17.65 ± 5.29	18.66 ± 4.12	*t*: −0.566; *p*: 0.572
	RS	15.69 ± 5.67	16.80 ± 5.11	*t*: −0.730; *p*: 0.466	15.66 ± 5.64	18.22 ± 5.16	*t*: −1.334; *p*: 0.184
	PSR	19.68 ± 6.09	18.26 ± 5.72	*t*: 0.873; *p*: 0.383	19.69 ± 6.05	17.11 ± 6.21	*t*: 1.252; *p*: 0.212
	SA	11.66 ± 4.34	10.86 ± 4.54	*t*: 0.685; *p*: 0.494	11.66 ± 4.33	10.22 ± 4.79	*t*: 0.977; *p*: 0.330
	DCP	14.95 ± 5.34	15.40 ± 5.15	*t*: −0.311; *p*: 0.756	14.98 ± 5.30	15.11 ± 5.90	*t*: −0.072; *p*: 0.943
	PNPS total	79.71 ± 22.97	78.86 ± 20.99	*t*: 0.139; *p*: 0.890	79.66 ± 22.83	79.33 ± 23.32	*t*: 0.043; *p*: 0.966

*Note:* *t*: independent sample *T*‐test value, *p*: significance level, XX ± YY: mean ± standard deviation. Significant *p* values are shown in bold, *p* < 0.05.

Abbreviations: ANS, aesthetic nursing skill; DCP, Desire to Continue the Profession; ELAS, ethical and legal issues application skill; EOP, Ethically Oriented Practice; GA, General Ability; HBSSNP, Humanistic Behaviour Skill Scale in Nursing Practice; HNCS, Holistic Nursing Competence Scale; NCS, nursing communication skill; NCT, Nursing Care in the Team; PAS, psychological adaptation skill; PCS, practical care skill; PD, professional development; PE, professional emotion; PEM, Personnel Education and Management; PNPS, Profession Nursing Pride Scale; PSR, problem‐solving role; RS, role satisfaction; SA, self‐achievement.

### 3.2 Association Between Socio‐Demographic Characteristics and Scale Scores

ANSs and Desire to Continue the Profession sub‐dimension scores of women nurses were statistically significantly higher than those of men (*p* < 0.05). No statistically significant difference was found between the nurses' marital status, education level and the scale sub‐dimensions and total score (*p* > 0.05). Nurses' scores showed that the Professional emotional PSR of nurses whose income was equal to their expenses was statistically significantly higher than that of nurses whose income was less than their expenses (*p* < 0.05) (Table 1).

It was found that the total and sub‐dimension scores of HNCS, HBSSNP and PNPS of nurses who willingly chose the nursing profession and liked their profession were statistically significantly higher (*p* < 0.05). The results showed that the total and sub‐dimension scores of the nurses working at night were statistically significantly higher than those working during the day (*p* < 0.05). In addition, it was determined that the mean scores of the sub‐dimension of RS and the total score of PNPS of the nurses working at night and daytime were statistically significantly higher than the nurses working during the day (*p* < 0.05). The Ethical and Legal Issues Implementation Skill, ANS sub‐dimension score of nurses working in internal services and the Ethical and Legal Issues Implementation Skill, PSR sub‐dimension score of nurses working in surgical services were statistically significantly higher than nurses working in outpatient clinics (*p* < 0.05) (Table 2).

It was found that nurses who considered the individual being cared for as a whole in terms of bio‐psycho‐socio‐cultural aspects without discriminating religion, language and race had statistically significantly higher scores in GA, Ethics‐Oriented Practice, Nursing Care in Team, PD, PAS, ANS, PCS sub‐dimensions, total score of HNCS and HBSSNP. In addition, it was determined that the total and sub‐dimensions of HNCS, PAS, PCS and HBSSNP total score of nurses who established effective and healthy communication with the individual being cared for were statistically significantly higher (*p* < 0.05) (Table 3).

### 3.3 Total Scores From the Scales

As a result of this study, the mean ± standard deviation of the students' HNCS, HBSSNP and PNPS total scores were 170.48 ± 42.41 points, 121.35 ± 20.81 points and 79.65 ± 22.80 points, respectively. According to the findings, the nurses' holistic nursing competencies were above average, their humanistic behaviour skills in nursing practices were high, and their professional pride was below average (Table 4).

**TABLE 4:** Total and sub‐dimensional scores of nurses' HNCS, HBNPS, PNPS.

Scale and subscales		Minimum	Maximum	Mean	Standard deviation
HNCS	GA	7.00	49.00	34.76	8.54
	PEM	9.00	63.00	39.87	11.39
	EOP	9.00	63.00	43.43	11.98
	NCT	7.00	49.00	33.43	9.56
	PD	4.00	28.00	18.97	5.59
	HNCS total	36.00	252.00	170.48	42.41
HBSSNP	NCS	7.00	35.00	27.95	4.92
	PAS	4.00	20.00	15.73	3.21
	ELAS	7.00	35.00	28.70	5.38
	ANS	4.00	20.00	15.58	3.36
	PCS	7.00	49.00	33.37	9.53
	HBSSNP total	69.00	159.00	121.35	20.81
PNPS	PE	6.00	30.00	17.69	5.24
	RS	6.00	30.00	15.77	5.63
	PSR	6.00	30.00	19.58	6.06
	SA	4.00	20.00	11.61	4.35
	DCP	5.00	25.00	14.98	5.31
	PNPS total	27.00	135.00	79.65	22.80

Abbreviations: ANS, aesthetic nursing skill; DCP, Desire to Continue the Profession; ELAS, ethical and legal issues application skill; EOP, Ethically Oriented Practice; GA, General Ability; HBSSNP, Humanistic Behaviour Skill Scale in Nursing Practice; HNCS, Holistic Nursing Competence Scale; NCS, nursing communication skill; NCT, Nursing Care in the Team; PAS, psychological adaptation skill; PCS, practical care skill; PD, professional development; PE, professional emotion; PEM, Personnel Education and Management; PNPS, Profession Nursing Pride Scale; PSR, problem‐solving role; RS, role satisfaction; SA, self‐achievement.

### 3.4 Correlation Between Scale Scores

A statistically significant positive correlation was observed between the HNCS and the Humanistic Behaviour in Nursing Practice Scale (*p* < 0.001). Additionally, a weak positive correlation was found between the HNCS and the PNPS. A very weak positive correlation was also identified between the Humanistic Behaviour in Nursing Practice Scale and the PNPS (*p* < 0.05) (Table 5).

**TABLE 5:** The correlation between the total scores of the nurses' HNCS, HBSSNP, PNPS.

Scales		HNCS total	HBSSNP total	PNPS total
HNCS total	*r*	1	0.725^a^	0.338^a^
	*p*		**< 0.001**	**< 0.001**
HBSSNP total	*r*	0.725^b^	1	0.184^a^
	*p*	**< 0.001**		**0.006**
PNPS total	*r*	0.338^a^	0.184^c^	1
	*p*	**< 0.001**	**0.006**	

*Note:* *r*: Pearson correlation analysis coefficient, *p*: significance value. Significant *p* values are shown in bold, *p* < 0.05.

Abbreviations: HBSSNP, Humanistic Behaviour Skill Scale in Nursing Practice; HNCS, Holistic Nursing Competence Scale; PNPS, Profession Nursing Pride Scale.

^a^ Weak level correlation.

^b^ High level correlation.

^c^ Very weak correlation.

### 3.5 Findings Related to the Regression Model Including the HNCS and Independent Variables

In Table 6, the HNCS was analysed univariately with the independent variables of age, gender, marital status, income status, educational status, willingly choosing the profession, working style, liking the profession, the service clinic worked in, treating the individual cared for as a whole, and establishing effective and healthy communication. When Table 6 was analysed, it was found that age, gender, marital status, income, and educational status were not statistically significant (*p* > 0.05). It was determined that the status of choosing the profession willingly, working style, liking the profession, the service clinic worked in, considering the individual being cared for as a whole, and establishing effective and healthy communication were statistically significant (*p* < 0.05) (Table 6).

**TABLE 6:** Univariate and multiple models established with the Holistic Nursing Competence Scale.

Variable	$\beta_{i}$	SE	*t*	*p*	CI
Univariate					
Age	−1.07	0.71	−1.51	0.133	−2.46 to 0.33
Gender	6.15	5.71	1.08	0.282	−5.09 to 17.39
Marital status	4.09	6.68	0.61	0.540	−9.06 to 17.26
Income status	3.82	4.40	0.87	0.387	−4.86 to 12.49
Education status	5.17	5.47	0.95	0.346	−5.61 to 15.94
Status of voluntary choice of nursing profession	−15.79	5.85	−2.70	**0.007**	−27.32 to −4.26
Mode of operation	10.18	3.71	2.74	**0.007**	2.86 to 17.49
Occupation affection status	−20.09	5.91	−3.40	**0.001**	−31.73 to −8.44
Worked service	−4.88	2.41	−2.01	**0.045**	−9.59 to −0.11
Considering the cared individual as a whole in terms of bio‐psycho‐psycho‐socio‐cultural aspects without discriminating religion, language, race	−26.39	11.23	−2.35	**0.020**	−48.51 to −4.27
Effective and healthy communication with the individual being cared for	−51.21	14.05	−3.65	**< 0.001**	−78.90 to −23.52
Multiple					
Status of voluntary choice of nursing profession				0.222	
Mode of operation	9.41	3.55	2.65	**0.009**	2.41 to 16.40
Occupation affection status	−17.50	5.73	−3.05	**0.003**	−28.80 to −6.21
Worked service				0.391	
Considering the cared individual as a whole in terms of bio‐psycho‐psycho‐socio‐cultural aspects without discriminating religion, language, race				0.842	
Effective and healthy communication with the individual being cared for	−46.12	13.67	−3.37	**0.001**	−73.06 to −19.18

*Note:* Significant *p* values are shown in bold, *p* < 0.05.

Abbreviations: CI, confidance interval; SE, standart error; *β*_*i*_, beta.

The HNCS score of those who did not choose the profession willingly decreased 15.79 times compared to those who did; the HNCS score of those who worked at night increased 10.18 times compared to those who worked both day and night; the HNCS score of those who did not like the profession decreased 20.09 times; the HNCS score decreased 4.88 times according to the service categories (intensive care, internal service, surgical service, emergency service, outpatient clinic); the HNCS score decreased 26.39 times more than those who did not treat the individual cared for as a whole; and the HNCS score decreased 51.21 times more than those who did not establish effective and healthy communication (Table 6).

In Table 6, multiple regression analysis was performed on the HNCS scale with the independent variables of willingly choosing the profession, working style, liking the profession, the service clinic worked in, treating the individual under care as a whole, and establishing effective and healthy communication. When Table 6 was analysed, it was found that in the established model, the status of choosing the profession willingly, the service clinic where the patient worked, and the status of considering the individual under care as a whole were not statistically significant (*p* > 0.05). However, in the established multiple regression model, it was found that working style, liking the profession, and establishing effective and healthy communication were statistically significant (*p* < 0.05) (Table 6).

It was found that the HNCS score increased 9.41 times for those who worked at night compared to those who worked during the day, for those who worked both day and night compared to those who worked at night, for those who did not like the profession compared to those who did, the HNCS score decreased 17.50 times, and for those who did not communicate effectively and healthily compared to those who did, the HNCS scale score decreased 46.12 times (Table 6).

### 3.6 Findings Related to the Regression Model Including the Scale of Humanistic Behaviour Skills in Nursing Practice and Independent Variables

In Table 7, the scale of HBSSNP was analysed univariately with the independent variables of age, gender, marital status, income status, educational status, willingly choosing the profession, working style, liking the profession, service clinic worked in, treating the individual cared for as a whole, and establishing effective and healthy communication. When Table 7 was analysed, it was found that age, gender, marital status, income, and educational status were not statistically significant (*p* > 0.05). It was determined that the status of choosing the profession willingly, working style, liking the profession, the service clinic worked in, considering the individual being cared for as a whole, and establishing effective and healthy communication were statistically significant (*p* < 0.05) (Table 7).

**TABLE 7:** Univariate and multiple models established with the humanistic behaviour skills in Nursing Practice Scale.

Variable	$\beta_{i}$	SE	*t*	*p*	CI
Univariate					
Age	−0.74	0.35	−2.13	**0.035**	−1.42 to −0.05
Gender	−1.64	2.80	−0.58	0.560	−7.16 to 3.89
Marital status	2.96	3.27	0.91	0.366	−3.49 to 9.42
Income status	2.92	2.15	1.36	0.176	−1.32 to 7.17
Education status	−0.13	2.69	0.05	0.960	−5.43 to 5.16
Status of voluntary choice of nursing profession	−7.61	2.87	−2.65	**0.009**	−13.27 to −1.95
Mode of operation	3.74	1.83	2.04	**0.043**	0.12 to 7.35
Occupation affection status	−10.99	2.88	−3.81	**< 0.001**	−16.67 to −5.32
Worked service	−2.68	1.18	−2.27	**0.024**	−4.99 to −0.36
Considering the cared individual as a whole in terms of bio‐psycho‐psycho‐socio‐cultural aspects without discriminating religion, language, race	−14.17	5.49	−2.58	**0.011**	−25.00 to −3.35
Effective and healthy communication with the individual being cared for	−20.05	6.97	−2.88	**0.004**	−33.79 to −6.32
Multiple					
Age				0.166	
Status of voluntary choice of nursing profession				0.346	
Mode of operation				0.058	
Occupation affection status	−10.26	2.86	−3.59	**< 0.001**	−15.89 to −4.62
Worked service				0.052	
Considering the cared individual as a whole in terms of bio‐psycho‐psycho‐socio‐cultural aspects without discriminating religion, language, race				0.292	
Effective and healthy communication with the individual being cared for	−17.60	6.82	−2.58	**0.011**	−31.05 to −4.15

*Note:* Significant *p* values are shown in bold, *p* < 0.05.

Abbreviations: CI, confidance interval; SE, standart error; *β*_*i*_, beta.

It was determined that the HBSSNP score of those who did not choose the profession willingly decreased 7.61 times compared to those who did, and the HBSSNP score of those who worked at night increased 3.74 times compared to those who worked both day and night. The HBSSNP score of those who do not like the profession decreased by 10.99 times; the HBSSNP score decreased by 2.68 times according to the service categories (intensive care, internal service, surgical service, emergency service, outpatient clinic). Sixty‐eight times, the HBSSNP score decreased 14.17 times more than those who did not consider the individual receiving care as a whole, and the HBSSNP score decreased 20.05 times more than those who did not establish effective and healthy communication (Table 7).

In Table 7, multiple regression analysis was performed on the scale of HBSSNP with the independent variables of age, willingly choosing the profession, working style, liking the profession, service clinic, considering the individual under care as a whole, and establishing effective and healthy communication. When Table 7 was analysed, it was found that age, willingly choosing the profession, working style, working service clinic and considering the individual under care as a whole were not statistically significant in the established model (*p* > 0.05). However, in the multiple regression model, it was found that the status of liking the profession and establishing effective and healthy communication were statistically significant (*p* < 0.05) (Table 7).

It was determined that the HBSSNP score of those who did not like the profession decreased 10.26 times less than those who liked the profession, and the HBSSNP score of those who did not establish effective and healthy communication decreased 17.60 times less than those who did (Table 7).

### 3.7 Findings Related to the Regression Model Including the PNPS and Independent Variables

In Table 8, the PNPS was analysed univariately with the independent variables of age, gender, marital status, income status, educational status, willingly choosing the profession, working style, liking the profession, service clinic, treating the individual under care as a whole and establishing effective and healthy communication. When Table 8 was analysed, it was found that age, gender, marital status, educational status, the service clinic worked in, treating the individual under care as a whole and establishing effective and healthy communication were not statistically significant (*p* > 0.05). Income status, willingly choosing the profession, working style and liking the profession were found to be statistically significant (*p* < 0.05) (Table 8).

**TABLE 8:** Univariate and multiple models established with the Pride in Nursing Profession Scale.

Variable	$\beta_{i}$	SE	*t*	*p*	CI
Univariate					
Age	−0.47	0.38	−1.24	0.216	−1.22 to −0.28
Gender	−4.92	3.06	−1.61	0.109	−10.95 to 1.10
Marital status	4.47	3.58	1.25	0.213	−2.59 to 11.52
Income status	6.37	2.33	2.73	**0.007**	1.78 to 10.97
Education status	2.78	2.94	0.95	0.345	−3.01 to 8.57
Status of voluntary choice of nursing profession	−9.34	3.13	−2.98	**0.003**	−15.52 to −3.17
Mode of operation	5.09	1.99	2.55	**0.012**	1.15 to 9.03
Occupation affection status	−10.87	3.18	−3.42	**0.001**	−17.13 to −4.61
Worked service	−0.65	1.30	−0.50	0.619	−3.22 to −1.92
Considering the cared individual as a whole in terms of bio‐psycho‐psycho‐socio‐cultural aspects without discriminating religion, language, race	−0.85	6.11	−0.14	0.890	−12.86 to −11.19
Effective and healthy communication with the individual being cared for	−0.34	7.78	−0.04	0.966	−15.66 to 14.99
Multiple					
Income status	4.95	2.30	2.15	**0.033**	0.42 to 9.48
Status of voluntary choice of nursing profession				0.005	
Mode of operation	4.59	1.94	2.36	**0.019**	0.76 to 8.42
Occupation affection status	−9.45	3.16	−2.99	**0.003**	−15.64 to −3.23

*Note:* Significant *p* values are shown in bold, *p* < 0.05.

Abbreviations: CI, confidance interval; SE, standart error; *β*_*i*_, beta.

It was determined that the score of the PNPS increased 6.37 times for those whose income was equal to expenses compared to those whose income was less than expenses, and for those whose income was more than expenses compared to those whose income was equal to expenses. It was found that the score of the PNPS decreased 9.34 times for those who did not choose the profession willingly, the score of the PNPS increased 5.09 times for those who worked at night compared to those who worked during the day, and for those who worked both day and night compared to those who worked at night, and the score of the PNPS decreased 10.87 times for those who did not like the profession compared to those who liked it (Table 8).

In Table 8, multiple regression analysis was performed with the independent variables of income status, willingly choosing the profession, working style and liking the profession in the PNPS. When Table 8 was examined, it was found that the status of choosing the profession willingly was not statistically significant in the established model (*p* > 0.05). However, in the established multiple regression model, income status, working style and liking the profession were found to be statistically significant (*p* < 0.05) (Table 8).

It was determined that the score of the PNPS increased 4.95 times for those whose income was equal to expenses compared to those whose income was less than expenses, and for those whose income was more than expenses compared to those whose income was equal to expenses. It was determined that the score of the PNPS increased 4.59 times for those whose working style was night compared to day, for those who worked both day and night compared to night, and the score of the PNPS decreased 9.45 times for those who disliked the profession compared to those who liked it (Table 8).

### 3.8 Findings on the Effect of Nurses' Holistic Nursing Competency Levels and Humanistic Behaviour Skills on Their Professional Pride

In the multiple regression analysis conducted to investigate the effect of nurses' holistic and humanistic behavioural skills on professional pride, the model established was statistically significant (*F* = 15.413 Model (*p*) < 0.001). The Durbin‐Watson value between 1.5 and 2.5 indicates no autocorrelation problem in the model (DW = 1.799) (Field 2024). A statistically significant effect was found in the regression analysis performed to determine how holistic and humanistic behavioural skills in nursing predicted the level of professional pride (*F* = 15.413; *p* < 0.001). The holistic and humanistic behavioural skills in nursing explained 12.2% of the change in professional pride (*R* ^2^ = 0.122) (Table 9).

**TABLE 9:** The effect of nurses' holistic and humanistic behavioural skills on professional pride.

Dependent variable	Independent variable	*β*	*t*	*p*	*F*	Model (*p*)	*R* ^2^	DW
PNPS	Constant value	57.249	6.726	**< 0.001**	15.413	**< 0.001**	0.122	1.799
	HNCS	0.232	4.724	**< 0.001**				
	HBSSNP	−0.142	−1.413	**< 0.001**				

*Note:* Significance, *β* value: it allows us to understand the direction and strength of the relationship between the independent variables in the model and the dependent variable, *t* value: tests whether the effect of each independent variable on the dependent variable is significant, *p*: significance value, *F* value: it is used to test whether the independent variables of the model have a significant effect on the dependent variable, adjusted *R* ^2^: explanatory power of the model. Significant *p* values are shown in bold, *p* < 0.05.

Abbreviations: DW, Durbin–Watson; HBSSNP, Humanistic Behaviour Skill Scale in Nursing Practice; HNCS, Holistic Nursing Competence Scale; PNPS, Profession Nursing Pride Scale.

## 4 Discussion

The study examined the effect of nurses' holistic nursing competency levels and humanistic behavioural skills on their professional pride levels. It aims to reveal how nurses' commitment to their profession and pride are affected when they take a holistic, human‐centered approach to patient care. The findings obtained were discussed in this context by comparing them with the existing studies in the literature.

### 4.1 Discussion of Findings Related to Sociodemographic Characteristics

Among the nurses, the ANS and Desire to Continue the Profession sub‐dimension scores of the women were statistically significantly higher than those of the men. Gender roles refer to socially imposed behaviour, thought, and lifestyle patterns for men and women. According to gender roles, men should be interested in jobs requiring power, housekeeping, and repair work, and women should be interested in housework, child care, aesthetic skills and cleaning work (Eryilmaz 2020). This situation may have led to women's caregiving roles and aesthetic skills in nursing practices. Accordingly, it can be said that there is an increase in professional skills and the DCP. In this direction, it is necessary to reduce the effect of sexist approaches and gender stereotypes in the nursing profession. For this purpose, it is recommended that a gender equality approach be adopted in nursing education programmes, policies that offer equal job opportunities should be targeted, and campaigns should be implemented to change social perception.

The results showed that the professional emotional PSR of the nurses whose income was equal to their expenses was statistically significantly higher than those whose income was less. This may indicate that nurses with a relatively more balanced income have more developed PEs and problem‐solving skills. The literature states that a low‐income level leads to negative job satisfaction levels and other skills (Fallahi and Mehrad 2015). This situation emphasises that income balance and financial welfare level in nursing also affect PE and problem‐solving skills. To reduce this negative effect and increase PE and problem‐solving skills, financial support services should be provided to nurses, fairer wage policies should be developed and attempts should be made to reduce other sources of stress.

The statistically significantly higher total and sub‐dimension scores of HNCS, HBSSNP and PNPS of nurses who willingly chose the nursing profession and liked their profession indicate that nurses' commitment to their profession and their working approach based on ethical values are more substantial. Preferring and liking the profession willingly is a factor that increases both professional commitment and openness to development. These characteristics contribute to nurses adopting humanitarian and ethical values and make the profession more professional (Tuna and Sahin 2021). The high level of holistic nursing competence, humanistic, behavioural skills, and professional pride of individuals who willingly choose the nursing profession increases the quality of health services. It contributes positively to the social perception of the profession.

We observed that the total and sub‐dimension scores of the holistic nursing approach of nurses working at night were statistically significantly higher than those of nurses working during the day. In addition, it was determined that the RS and pride in the nursing profession of nurses working mixed (day and night) were statistically significantly higher than nurses working during the day. This may be attributed to the increase in holistic care competencies and RS of nurses working on the night shift due to working in a calmer environment and providing more comprehensive nursing care. The increase in RS may also have increased the level of pride in the profession. It is stated in the literature that a well‐organised shift system will increase professional and personal satisfaction (Rosa et al. 2019). Therefore, making night or mixed shifts more attractive, receiving regular feedback on shifts and implementing flexible shift systems that encourage nurses to maintain work‐life balance will increase the quality of care, satisfaction and pride in the profession.

It was the data demonstrated that the application of ethical and legal issues, ANSs and PSRs of nurses working in internal and surgical wards was statistically significantly higher than that of nurses working in outpatient clinics. It is thought that nurses working in outpatient clinics spend less time with patients and, therefore, have less need for ethical processes and problem‐solving skills. In internal and surgical wards, patient care is handled in a more complex and holistic manner. These patients require more personalised and aesthetic care. Benner's (1984) Clinical Skills Theory states that as clinical experience increases, ethical, legal, aesthetic and problem‐solving skills also increase (Tilden and Tilden 1985). The higher level of these skills of nurses working in more intensive clinics may be associated with this situation. In this context, it is recommended that training should be organised to improve the problem‐solving skills of nurses, especially outpatient clinic nurses, with more complex clinical processes and ethical and legal issues.

In this study, nurses who treat the individual being cared for as a whole in terms of biological, psychological, social, and cultural aspects without discriminating against religion, language and race have significantly higher GA, Ethics‐Oriented Practice, NCT, PD, PAS, ANS, PCS, Holistic Nursing Approach, and Humanistic Behaviour Skill. These results reveal that nurses who treat the patient holistically are more humanistic, have aesthetic skills, and have high PD and team cohesion. The literature shows that the holistic nursing approach increases interprofessional communication (Talebi 2023), and the aesthetic approach strengthens the hope and belief of the cared individual (Muslu and Ozsoy 2017). Therefore, for more humane and respectful care, the quality of care needs to approach all individuals equally without discrimination. In this context, seminars and training can be organised to support the PD of nurses. In addition, it was found that the holistic nursing competence, psychological adjustment, practical care, and humanistic behaviour skills of nurses who established effective and healthy communication with the individual being cared for were significantly higher. These findings are parallel with the literature, and it was determined that communication skills significantly affect nursing care quality. Effective and healthy communication constitutes the basic building block of nursing practices and means quality care (Wafa'a et al. 2024). Quality care contributes to healthier psychological adjustment processes and more humanistic care. In line with this study and the literature, it may be recommended to establish support systems to improve nurses' communication skills in both education and clinical practice processes. Cultural sensitivity training can be organised especially to communicate effectively and healthily with individuals from different cultures. In addition, continuous training and feedback systems can be developed to improve psychological adaptation and PCSs.

### 4.2 Discussion of Total Scale Scores

As a result of this study, the findings indicated that nurses' holistic nursing competence levels and humanistic behaviour skills in nursing practices were high. In contrast, their professional pride levels were low. When the literature was examined, it was determined that the holistic nursing competencies of nursing intern students were high in the study by Guler and Betul (2022), and the humanistic behaviour skills of nurses were high in the survey by Mercimek (2022), and these findings were in parallel with our research results (Guler and Betul 2022; Mercimek 2022). In addition, the low professional pride levels of nurses in this study may be associated with the intensive workload, stressful working conditions, and low social image of the nursing profession (Mauno et al. 2016). These findings reveal the need to improve professional pride in nurses. For this purpose, improving the working conditions of nurses, promoting the nursing profession correctly in social media channels, emphasising its importance, and increasing its prestige in society can prevent low professional pride and social perception. Strengthening the professional image with the studies carried out in this context will increase professional pride and thus positively affect job satisfaction, satisfaction, and quality of care.

### 4.3 Discussion of Findings Related to the Relationship Between Scales

This study observed that as the nurses' holistic nursing competence level and humanistic behaviour skills increased, their professional pride also increased. In other words, when nurses exhibit a more comprehensive (holistic) and human‐oriented (humanist) approach to patient care, their commitment to their profession and pride also increase. In other words, some of the professional pride of nurses can be explained by how holistic and human‐centered they are in care. This shows that professional pride depends on external factors and nurses' professional skills and attitudes. In this context, to increase the professional pride of nurses, holistic and humanistic approaches should be included more in educational programmes, PD should be supported, work environments should be improved and the achievements of nurses should be recognised more at the social level.

## 5 Strengths and Limitations

This study examined the professional pride levels of nurses by considering both holistic and humanistic approaches. This comprehensive approach offers an innovative contribution by revealing the relationship between these variables, which are generally considered independently in the literature. In addition, it comprehensively examined the effects of nurses' professional preferences, working conditions and individual characteristics on professional pride. This provides a broader contextualisation of the findings for the nursing profession.

One limitation of this study is the lack of a minimum professional experience requirement for participants. While the inclusion criteria ensured active employment as a nurse, differences in professional experience may have influenced the findings. Future studies should consider stratifying participants based on their years of experience to better understand its potential impact on the results. The findings of this study can be generalised only to the nurses working in the institution where the research was conducted because the research was conducted only with the nurses in that institution. The study was conducted using a cross‐sectional design. This makes it possible to identify the causal relationship between the variables rather than explaining it explicitly. The proportion of male nurses among the participants was low. This may impose limitations in assessing the effect of gender differences. Finally, the data were collected through scales filled out by the participants based on their own perceptions. This may lead to subjective biases such as social desirability bias. One of the limitations of this study is that, although multiple regression analysis was conducted, potential confounding factors were not fully accounted for in the model. While the analysis provides insights into the relationships between holistic nursing competencies, humanistic behaviour skills and professional pride, the absence of a comprehensive adjustment for all possible confounders may limit the accuracy of causal inferences. Future research should consider more advanced statistical models that incorporate a broader range of confounding variables to enhance the robustness of the findings.

## 6 Conclusions

It was found that nurses' holistic nursing competencies and humanistic behavioural skills in nursing practices were above average, and their professional pride was below average. In addition, it was found that nurses' holistic and humanistic approaches explained 12.2% of the change in professional pride level. These findings emphasise the importance of nurses' competencies and commitment to their profession for the development of professional pride. In this context,

- Training and seminars can be organised in nursing education institutions and hospitals to support PD.
- Organising cultural competence training for nursing students and nurses can increase their sensitivity towards individuals with different religions, languages and races.
- Improving nurses' workload, working hours and working conditions can positively affect their professional satisfaction and pride in the profession.
- A holistic and humanistic perspective can be gained in nursing practices with the guidance of competent and well‐equipped nurses for newly graduated nurses.
- Activities can be organised to strengthen communication and cooperation within the team.
- Career development for nurses' interests can be supported to strengthen the level of professional pride in nursing.
- Continuing education and PD programmes should be encouraged to improve nurses' professional skills and humanistic approaches.
- Professional motivation can be increased by rewarding and appreciating achievements in nursing practices.
- Social media platforms can be used to announce the importance of the nursing profession and its contributions to society, strengthening its commitment and image.
- By creating platforms where the community can share its opinions and suggestions regarding nursing care, nurses can feel that their work is valuable.
- In addition, ensuring peace of work, improving employment conditions, improving the financial return of the profession, planning a manageable workload, and establishing a fair wage distribution can increase nurses' motivation and reinforce their loyalty and pride in the profession.

## 7 Relevance to Clinical Practice

This study emphasises the importance of a humanistic and holistic approach in nursing practice and addresses professional pride. Humanistic, holistic approach and professional pride, important concepts in clinical practice areas, significantly affect care behaviours.

## Author Contributions

S.K., A.E., and M.M. contributed to the idea/concept, design, supervision, consultancy, data collection and processing, analysis and interpretation, literature review, manuscript writing, references, and critical review of the manuscript.

## Consent

Written informed consent was obtained from the students participating in the study.

## Conflicts of Interest

The authors declare no conflicts of interest.

## Data Availability

The data that support the findings of this study are available from the corresponding author upon reasonable request.
